# Supplementary material for: Efficient manipulation of gene dosage in human iPSCs using CRISPR/Cas9 nickases
Source: Commun Biol. 2021 Feb 12;4:195. doi: 10.1038/s42003-021-01722-0 (PMC7881037; doi:10.1038/s42003-021-01722-0)
Supplement: Supplementary file 2 — Supplementary Information [file 42003_2021_1722_MOESM2_ESM.pdf]

Supplementary Figure 1

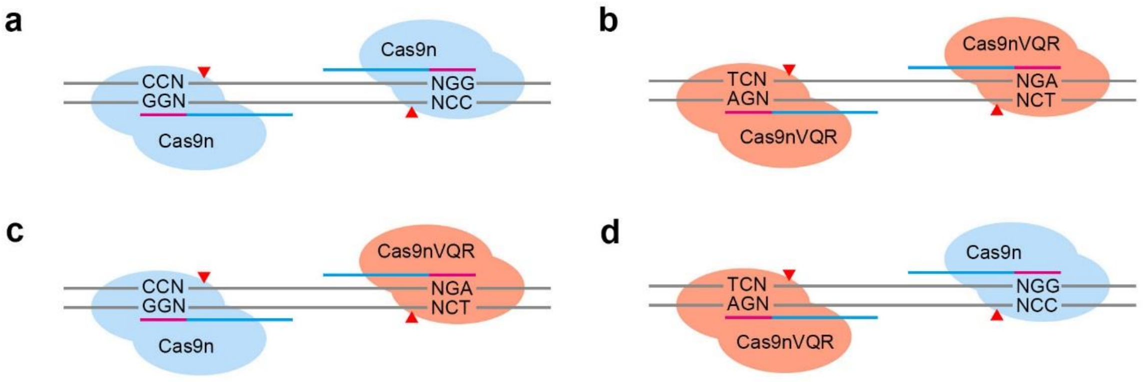

e

Cas9 nickase configuration	Binding motif	Number of targetable human genomic sites	Coverage of human genomic sites (of 3,209,286,105 total)
Cas9n	CC-$N_{42\text{-}62}$-GG	1,865,848,005	58.1%
Cas9nVQR	TC-$N_{42\text{-}62}$-GA	2,561,221,144	79.8%
Cas9nVQR & Cas9n	YC-$N_{42\text{-}62}$-GR	2,898,477,273	90.3%

Supplementary Figure 1. Increasing the genome targetability of double-nicking genome editing by using wild-type and engineered Cas9 nickases. A Cas9 nickase variant, Cas9nVQR (D1135V/R1335Q/T1337R), with altered protospacer-adjacent motif (PAM) specificity for the NGA sequence was generated. In addition to the existing configuration of paired Cas9 nickases (Cas9n) (a), Cas9nVQR can function alone (b) or together with Cas9n (c, d) to generate two adjacent DNA nicks. (e) Genome-wide coverage analysis of human genomic sites targetable by various Cas9 nickase configurations. In contrast to wild-type Cas9 nuclease, these configurations aim to expand the number of sites that can be edited by double-nicking genome editing and increase precision. Y: C or T, R: G or A, N: any base.

Supplementary Figure 2

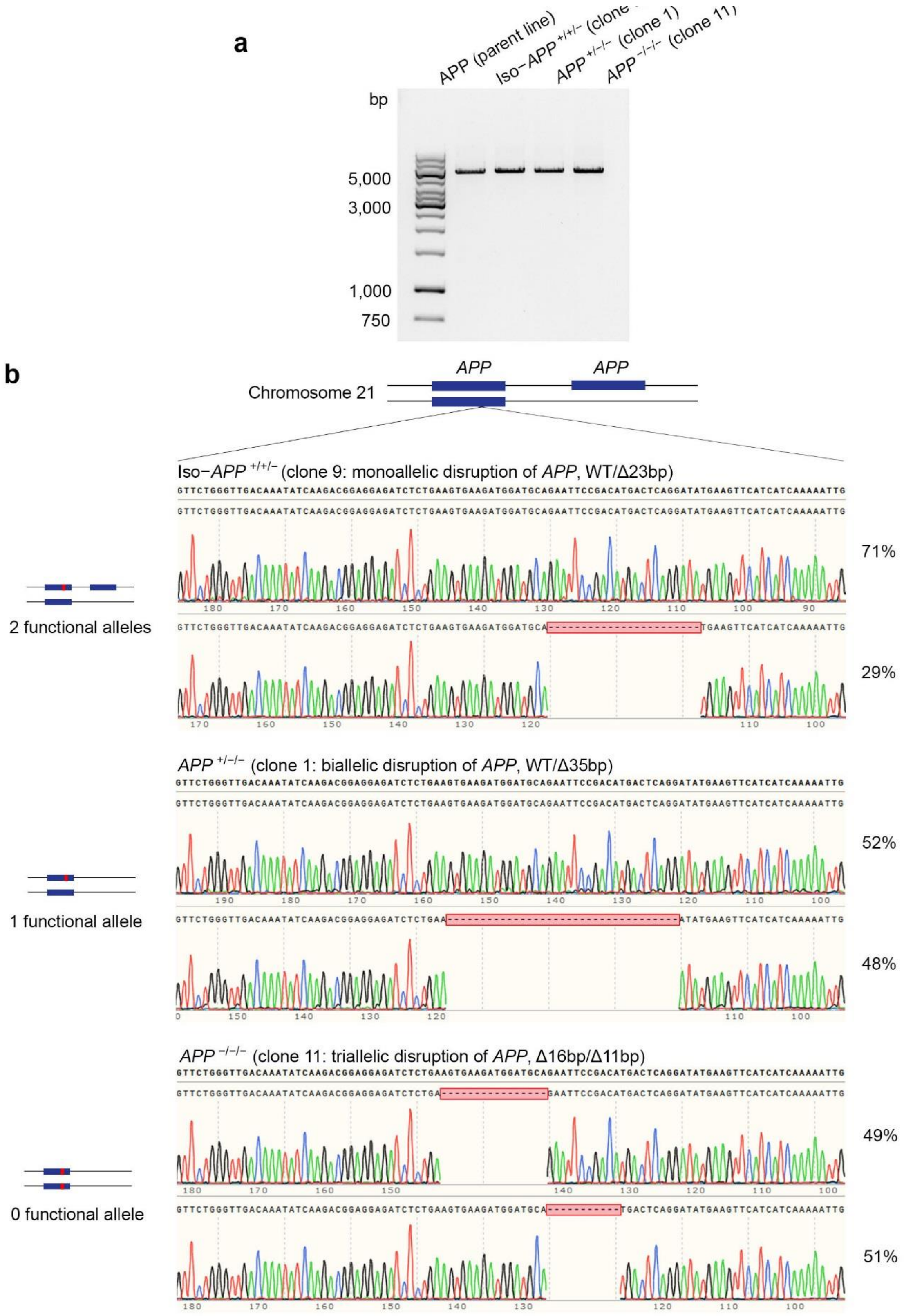

Supplementary Figure 2. Characterization of gene-edited human induced pluripotent stem cells. (a) Gel electrophoresis of the 5.2-kb PCR amplicons surrounding *APP* exon 16 detected no large deletions in gene-edited human induced pluripotent stem cell (iPSC) lines. (b) Sanger sequencing of the 1.2-kb PCR amplicons surrounding *APP* exon 16 detected specific deletion mutations in gene-edited iPSC lines. The ratio of frameshift deletions among the wild-type sequences indicated the simultaneous generation of iPSC lines with monoallelic, biallelic, or triallelic *APP* knockout. Iso: *APP* copy number-corrected isogenic cell line; WT: wild type.

Supplementary Figure 3

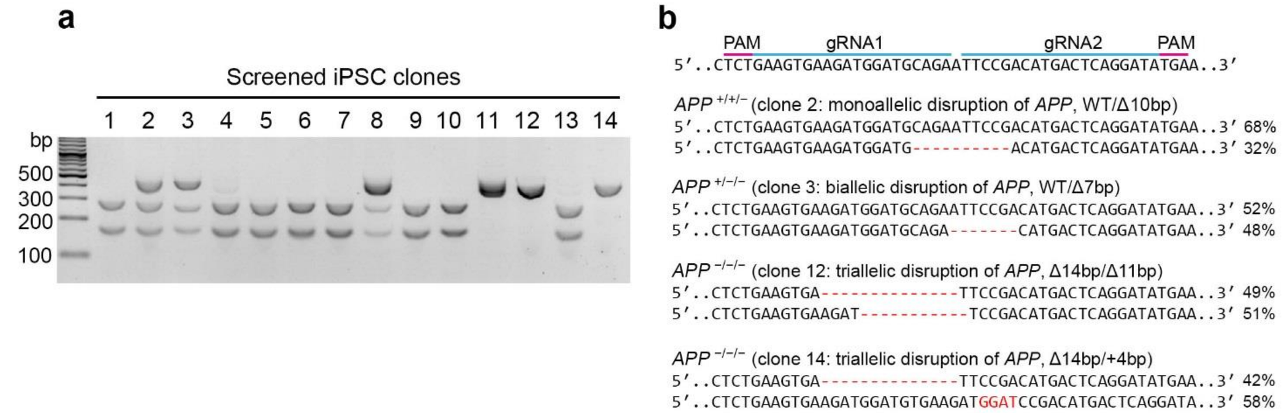

Supplementary Figure 3. Reproducible gene editing of *APP* copy number in human induced pluripotent stem cells by paired Cas9 nickases. (a) Screening of gene-edited human induced pluripotent stem cell (iPSC) clones by *Eco*RI digestion. (b) Specific deletion mutations detected in gene-edited iPSC clones by Sanger sequencing and next-generation deep sequencing. WT: wild type.

Supplementary Figure 4

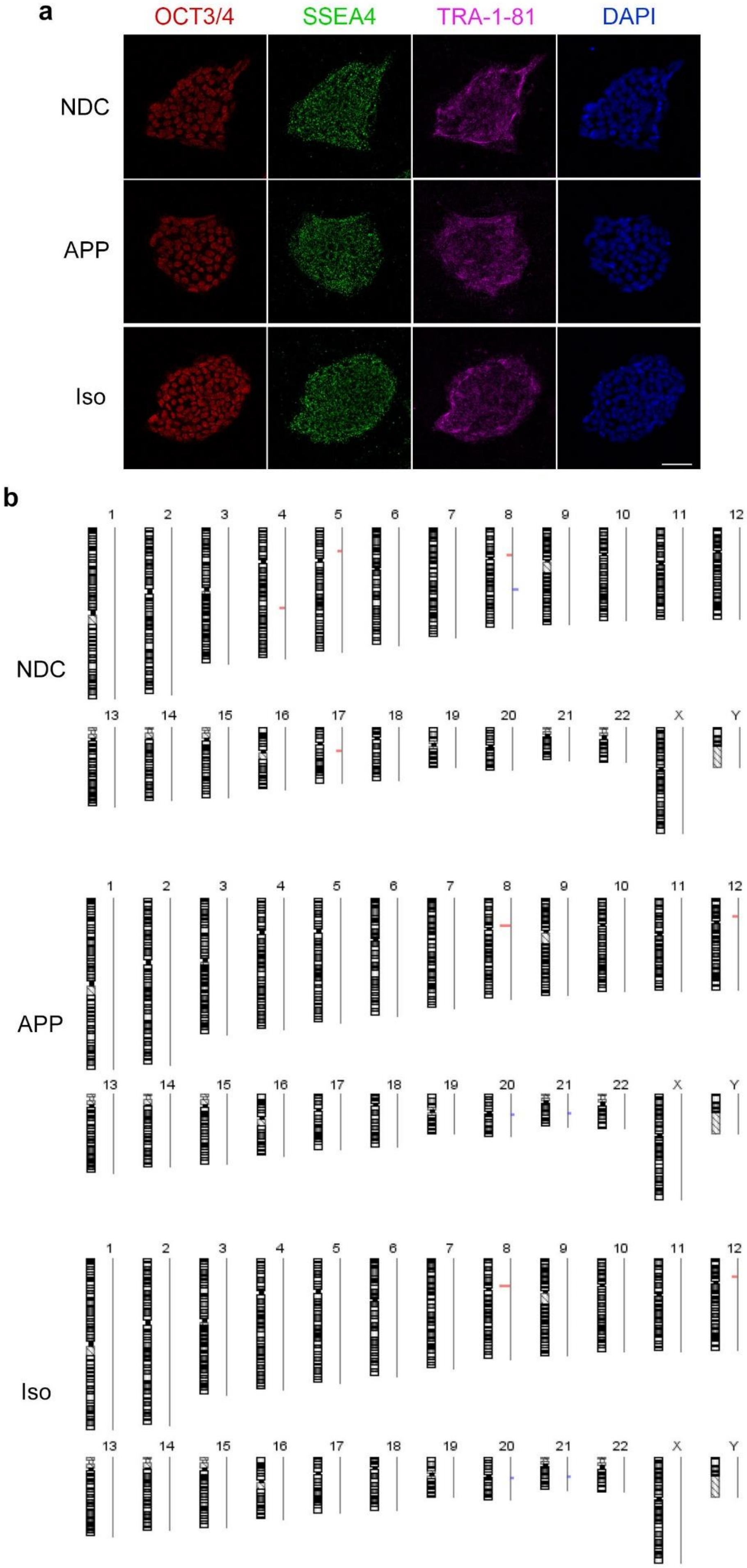

Supplementary Figure 4. Gene-edited human induced pluripotent stem cells maintain pluripotency and

a normal karyotype. (a) Human induced pluripotent stem cells (iPSCs) generated from a nondemented control (NDC), an Alzheimer’s disease (AD) patient with *APP* duplication (APP), and an *APP* copy number-corrected isogenic line (Iso) expressed the pluripotency markers OCT3/4 (red), SSEA4 (green), and TRA-1-81 (magenta). Scale bar: 50 μm. (b) Representative genomic overview of parental and corrected iPSCs. Blue and red bars indicate genomic regions exhibiting amplification and deletion, respectively.

Supplementary Figure 5

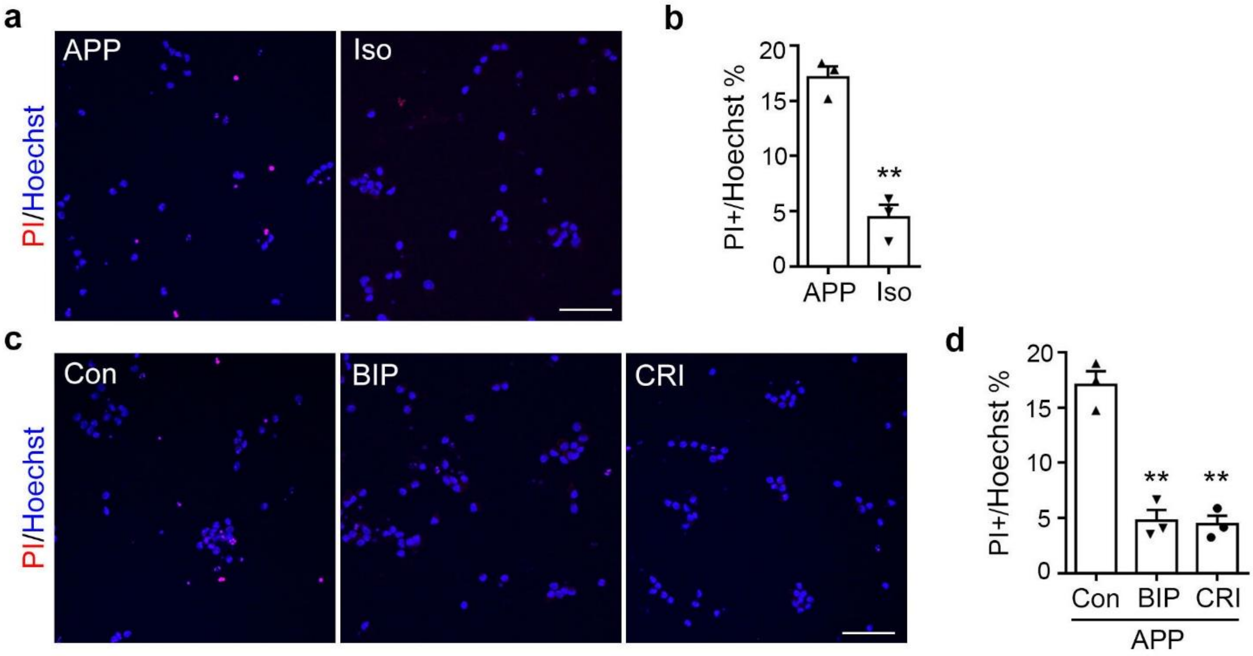

Supplementary Figure 5. Neuronal death is rescued in gene-corrected isogenic neurons. (a) Immunofluorescence images showing propidium iodide (PI)-positive apoptotic cells in *APP* copy number-corrected isogenic (Iso) neurons and *APP* duplication (APP) neurons at 28 days in vitro. Scale bar: 50 µm. (b) Percentage of PI-positive apoptotic cells in all neurons. The proportion of apoptotic cells was lower in Iso neurons than APP neurons. Values are mean ± SEM (n = 3 independent biological replicates per line; $**P < 0.01$ vs. APP line; Student's t-test). (c) Immunofluorescence images showing PI-positive cells in APP neurons at 28 days in vitro treated with vehicle control (Con), 100 µM BIP-V5 (Bax inhibitor peptide V5), or 5 µM CRI (cytochrome C release inhibitor) for 7 days. Scale bars: 50 µm. (d) Percentage of PI-positive apoptotic cells among all neurons. The proportion of apoptotic cells was lower in BIP-V5– or CRI-treated neurons than that in Con neurons. Values are mean ± SEM (n = 3 independent biological replicates per line; $**P < 0.01$ vs. Con neurons; Student's t-test).

Supplementary Figure 6

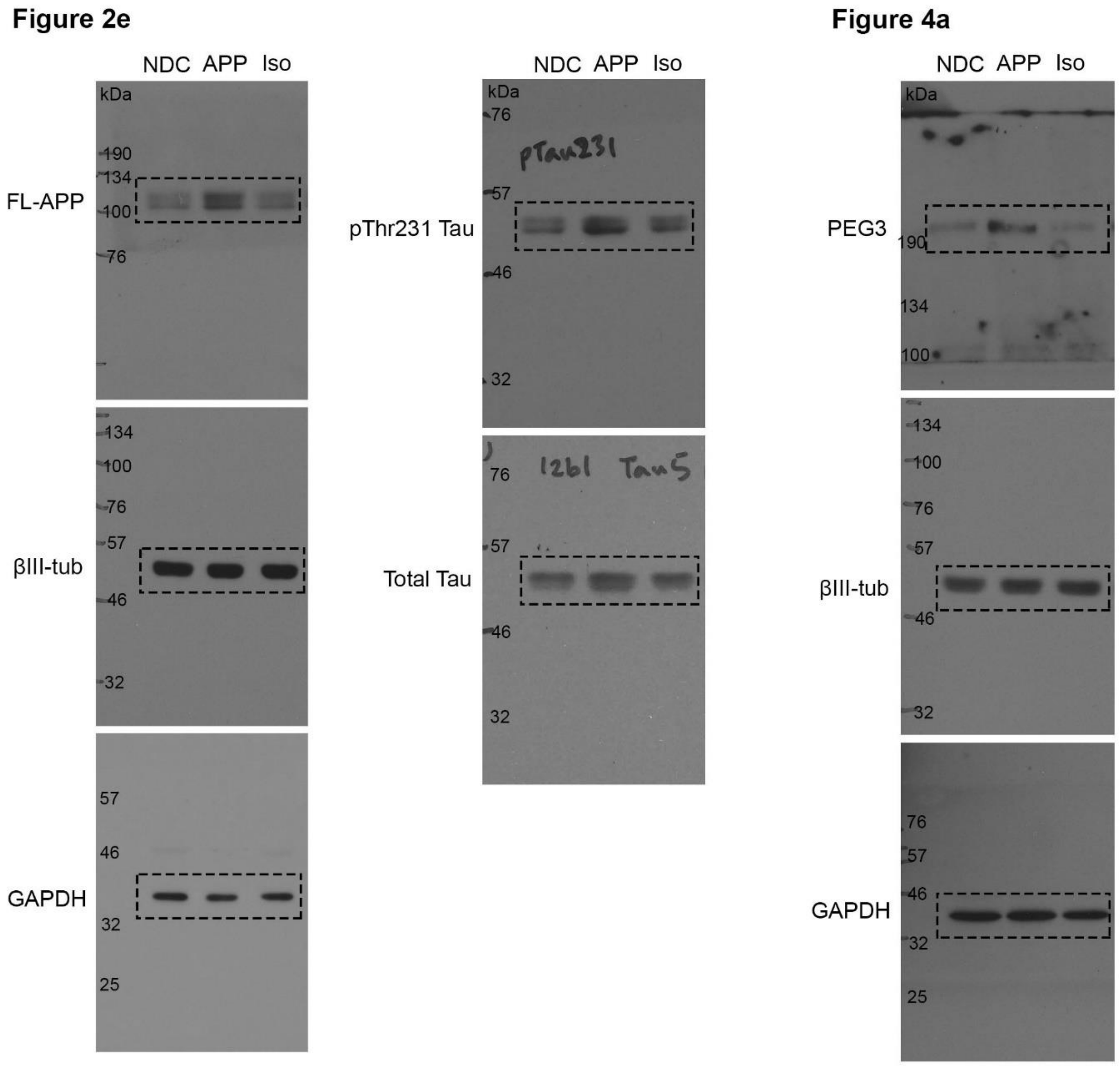

Supplementary Figure 6. Full-length blots corresponding to Figures 2e and 4a

Supplementary Table 1. Summary of Alzheimer's disease risk genes associated with a gene dosage effect[a]

Gene	Mutation type	Heterozygous KO iPSC	Homozygous KO iPSC	Molecular classification	Possible AD-related functions
APP	Duplication	Y[b, 1]	Y[2]	Integral membrane protein	APP expression
ABCA7	Noncoding	N[b]	N	ATP-binding cassette transporter	APP processing
ACE	Noncoding	N	N	Angiotensin-converting enzyme	Aβ degradation
ADAM10	Noncoding	N	N	Metalloprotease	APP processing
ADAMTS1	Noncoding	N	N	Metalloprotease	Unknown
BIN1	Noncoding	N	N	Endocytic adaptor	Tau toxicity
CASS4	Noncoding	N	N	Tyrosine kinase docking	Tau toxicity and axonal transport
CD2AP	Noncoding	N	N	Cytoskeletal organization	Tau toxicity
CD33	Noncoding	N	N	Surface receptor	Aβ clearance
CELF1	Noncoding	N	N	RNA-binding protein	Unknown
CLU	Noncoding	N	Y[3]	Extracellular chaperone	Aβ toxicity
CR1	Noncoding	N	N	Surface receptor	Aβ clearance
EPHA1	Noncoding	N	N	Receptor tyrosine kinase	Synaptic function
FERMT2	Noncoding	N	Y[4]	Extracellular matrix scaffolding	Tau toxicity
HLA-DRB1	Noncoding	N	N	Antigen presentation	Immune function
INPP5D	Noncoding	N	N	Phosphatidylinositol phosphatase	Immune function
IQCK	Noncoding	N	N	Calmodulin-binding domain	Unknown
MEF2C	Noncoding	N	N	Transcription factor	Synaptic function
MS4A6A	Noncoding	N	N	Transmembrane protein	Immune function
NME8	Noncoding	N	N	Thioredoxin domain	Unknown
PICALM	Noncoding	N	N	Endocytosis, clathrin assembly	Aβ clearance
PTK2B	Noncoding	N	N	Tyrosine kinase	Tau toxicity and synaptic function
SLC24A4	Noncoding	N	N	$Na^+/K^+/Ca^{2+}$ exchanger	Unknown
SORL1	Noncoding	N	Y[5]	Endocytic receptor/sorting	APP trafficking
SPI1	Noncoding	N	Y[6]	Transcription factor	Immune function
WWOX	Noncoding	N	N	Oxidoreductase	Tau toxicity
ZCWPW1	Noncoding	N	N	Zinc finger domain	Unknown

Abbreviations: Aβ: amyloid-beta; AD: Alzheimer's disease; APP: amyloid precursor protein; heterozygous KO: heterozygous or haplodeficient knockout; homozygous KO: homozygous or complete knockout; iPSC: induced pluripotent stem cell.

[a] Alzheimer's disease risk genes associated with copy number or noncoding variations suggested to alter gene expression levels or gene dosage are summarized from genetic meta-analyses[7, 8, 9].

[b] Y: reported in cited reference; N: not reported in cited reference.

Supplementary Table 2. Summary of the literature on the generation of induced pluripotent stem cell lines with homozygous and heterozygous knockout for Alzheimer's disease risk genes

Study (reference)	Days[a] required	Target gene	Manipulation steps	Footprint	Antibiotic selection	Heterozygous KO iPSC	Homozygous KO iPSC
Present study	~25	*APP*	1	None	None	Y	Y
Reference #1	~25	*APP*	1	Puro-TK	Puromycin	Y[b]	N[b]
Reference #2	~25	*APP*	1	None	None	N	Y
Reference #3	~25	*CLU*	1	GFP-neo	Neomycin	N	Y
Reference #4	~25	*FERMT2*	1	N/A	Puromycin & blasticidin	N	Y
Reference #5	~25	*SORL1*	1	None	None	N	Y
Reference #6	>50	*SPI1*	2	None	Puromycin	Y	Y
Reference #10	>50	*TREM2*	2	None	Hygromycin	Y	Y

Abbreviations: GFP-neo: GFP and floxed neomycin selection cassette; heterozygous KO: heterozygous or haplodeficient knockout; homozygous KO: homozygous or complete knockout; iPSC: induced pluripotent stem cell; N/A: not available/mentioned; Puro-TK: puromycin-thymidine kinase selection cassette.

[a] Days from the nucleofection of iPSCs to the generation of knockout iPSC lines (1-week expansion of sequencing-verified knockout iPSC clones).

[b] Y: reported in cited reference; N: not reported in cited reference.

Supplementary Table 3. Amplification and deletion carried by APP parent and edited induced pluripotent stem cell lines

iPSC lines	Chr	Start–stop (bp)	Size (bp)	Cytoband	Estimated copy number	Annotations (genes)
APP (parent line)<br>Iso–*APP*$^{+/+/-}$ (clone 9)<br>*APP*$^{+/-/-}$ (clone 1)<br>*APP*$^{-/-/-}$ (clone 11)	chr8	39,258,894–39,386,158	127,265	p11.22	0	*ADAM5P*, *ADAM3A*
	chr8	89,558,563–89,959,573	401,011	q21.3	4	
	chr12	26,491,063–26,608,568	117,506	p12.1–p11.23	1	*ITPR2*
	chr20	29,888,477–30,800,011	911,535	q11.21	3	*DEFB116*, *DEFB118*, *DEFB119*, *DEFB121*…
APP (parent line)<br>Iso-*APP*$^{+/+/-}$ (clone 9)	chr21	27,062,149–27,784,579	722,431	q21.3	3	*JAM2*, *ATP5J*, *GABPA*, *APP*

Abbreviations: Chr: chromosome; iPSC: induced pluripotent stem cell.

Supplementary References

1. Ovchinnikov DA, Korn O, Virshup I, Wells CA, Wolvetang EJ. The Impact of APP on Alzheimer-like Pathogenesis and Gene Expression in Down Syndrome iPSC-Derived Neurons. *Stem cell reports* **11**, 32-42 (2018).
2. Fong LK, *et al.* Full-length amyloid precursor protein regulates lipoprotein metabolism and amyloid-beta clearance in human astrocytes. *The Journal of biological chemistry* **293**, 11341-11357 (2018).
3. Robbins JP, *et al.* Clusterin Is Required for beta-Amyloid Toxicity in Human iPSC-Derived Neurons. *Front Neurosci* **12**, 504 (2018).
4. Sullivan SE, *et al.* Candidate-based screening via gene modulation in human neurons and astrocytes implicates FERMT2 in Abeta and TAU proteostasis. *Human molecular genetics* **28**, 718-735 (2019).
5. Knupp A, *et al.* Depletion of the AD risk gene SORL1 selectively impairs neuronal endosomal traffic independent of amyloidogenic APP processing. *bioRxiv*, 2020.2001.2006.896670 (2020).
6. Buchrieser J, James W, Moore MD. Human Induced Pluripotent Stem Cell-Derived Macrophages Share Ontogeny with MYB-Independent Tissue-Resident Macrophages. *Stem cell reports* **8**, 334-345 (2017).
7. Lambert JC, *et al.* Meta-analysis of 74,046 individuals identifies 11 new susceptibility loci for Alzheimer's disease. *Nature genetics* **45**, 1452-1458 (2013).
8. Jansen IE, *et al.* Genome-wide meta-analysis identifies new loci and functional pathways influencing Alzheimer's disease risk. *Nature genetics* **51**, 404-413 (2019).
9. Kunkle BW, *et al.* Genetic meta-analysis of diagnosed Alzheimer's disease identifies new risk loci and implicates Abeta, tau, immunity and lipid processing. *Nature genetics* **51**, 414-430 (2019).
10. Claes C, *et al.* Human stem cell-derived monocytes and microglia-like cells reveal impaired amyloid plaque clearance upon heterozygous or homozygous loss of TREM2. *Alzheimers Dement* **15**, 453-464 (2019).
