# Supplementary material for: Efficient manipulation of gene dosage in human iPSCs using CRISPR/Cas9 nickases
Source: Commun Biol. 2021 Feb 12;4:195. doi: 10.1038/s42003-021-01722-0 (PMC7881037; doi:10.1038/s42003-021-01722-0)
Supplement: Supplementary file 3 — Description of Supplementary Files [file 42003_2021_1722_MOESM3_ESM.pdf]

Description of Additional Supplementary Files

File name: Supplementary Data 1.

Description: Comparative genomic hybridization data of APP parent and edited induced pluripotent stem cell lines.

File name: Supplementary Data 2.

Description: Source data of the graphs and charts presented in the main figures.

File name: Supplementary Software 1.

Description: Custom code for genome-wide coverage analysis of Cas9 nickase configurations in the GRCh38 human reference genome.
