# Supplementary material for: Efficient manipulation of gene dosage in human iPSCs using CRISPR/Cas9 nickases
Source: Commun Biol. 2021 Feb 12;4:195. doi: 10.1038/s42003-021-01722-0 (PMC7881037; doi:10.1038/s42003-021-01722-0)
Supplement: Supplementary file 7 — Reporting Summary [file 42003_2021_1722_MOESM7_ESM.pdf]

nature research

Corresponding author(s): Nancy Ip

Last updated by author(s): Jan 7, 2021

Reporting Summary

Nature Research wishes to improve the reproducibility of the work that we publish. This form provides structure for consistency and transparency in reporting. For further information on Nature Research policies, see our Editorial Policies and the Editorial Policy Checklist.

Statistics

For all statistical analyses, confirm that the following items are present in the figure legend, table legend, main text, or Methods section.

n/a	Confirmed	
☐	☒	The exact sample size (n) for each experimental group/condition, given as a discrete number and unit of measurement
☐	☒	A statement on whether measurements were taken from distinct samples or whether the same sample was measured repeatedly
☐	☒	The statistical test(s) used AND whether they are one- or two-sided<br>*Only common tests should be described solely by name; describe more complex techniques in the Methods section.*
☒	☐	A description of all covariates tested
☒	☐	A description of any assumptions or corrections, such as tests of normality and adjustment for multiple comparisons
☐	☒	A full description of the statistical parameters including central tendency (e.g. means) or other basic estimates (e.g. regression coefficient) AND variation (e.g. standard deviation) or associated estimates of uncertainty (e.g. confidence intervals)
☐	☒	For null hypothesis testing, the test statistic (e.g. F, t, r) with confidence intervals, effect sizes, degrees of freedom and P value noted<br>*Give P values as exact values whenever suitable.*
☒	☐	For Bayesian analysis, information on the choice of priors and Markov chain Monte Carlo settings
☒	☐	For hierarchical and complex designs, identification of the appropriate level for tests and full reporting of outcomes
☒	☐	Estimates of effect sizes (e.g. Cohen's d, Pearson's r), indicating how they were calculated

Our web collection on statistics for biologists contains articles on many of the points above.

Software and code

Policy information about availability of computer code

Data collection	LAS X (Leica), V-PLEX Aβ Peptide Panel 1 (6E10) Kit (Meso Scale Discovery), illumina HiSeq.
Data analysis	ImageJ 1.51h, GraphPad Prism 6, CRISPResso2, HISAT2, StringTie, DESeq2, R, samtools, bedtools. Custom code for genome-wide coverage analysis of Cas9 nickase configurations in the GRCh38 human reference genome is available in Supplementary Software 1.

For manuscripts utilizing custom algorithms or software that are central to the research but not yet described in published literature, software must be made available to editors and reviewers. We strongly encourage code deposition in a community repository (e.g. GitHub). See the Nature Research guidelines for submitting code & software for further information.

Data

Policy information about availability of data

All manuscripts must include a data availability statement. This statement should provide the following information, where applicable:

- Accession codes, unique identifiers, or web links for publicly available datasets
- A list of figures that have associated raw data
- A description of any restrictions on data availability

The deep sequencing data are deposited in the NCBI Sequence Read Archive (SRA; accession code: PRJNA681926). The RNA sequencing data are deposited in the NCBI Gene Expression Omnibus (GEO; accession code: GSE160224). The comparative genomic hybridization data are available in Supplementary Data 1. Source data of the graphs and charts presented in the main figures are available in Supplementary Data 2.

April 2020

Field-specific reporting

Please select the one below that is the best fit for your research. If you are not sure, read the appropriate sections before making your selection.

☒ Life sciences ☐ Behavioural & social sciences ☐ Ecological, evolutionary & environmental sciences

For a reference copy of the document with all sections, see nature.com/documents/nr-reporting-summary-flat.pdf

Life sciences study design

All studies must disclose on these points even when the disclosure is negative.

Sample size	No statistical methods were used to predetermine sample size.
Data exclusions	No data were excluded from the study.
Replication	All findings were reproduced in 3 or 4 independent differentiation experiments with the same methodology.
Randomization	The differentiated neurons were stained with indicated antibodies and photos were randomly taken.
Blinding	The investigators were blinded to the identity of each sample during data collection and analysis.

Reporting for specific materials, systems and methods

We require information from authors about some types of materials, experimental systems and methods used in many studies. Here, indicate whether each material, system or method listed is relevant to your study. If you are not sure if a list item applies to your research, read the appropriate section before selecting a response.

Materials & experimental systems

n/a	Involved in the study	
☐	☒	Antibodies
☐	☒	Eukaryotic cell lines
☒	☐	Palaeontology and archaeology
☒	☐	Animals and other organisms
☒	☐	Human research participants
☒	☐	Clinical data
☒	☐	Dual use research of concern

Methods

n/a	Involved in the study	
☒	☐	ChIP-seq
☒	☐	Flow cytometry
☒	☐	MRI-based neuroimaging

Antibodies

Antibodies used	We used the following primary antibodies for immunofluorescence and western blot analysis: mouse anti–Oct-3/4 (C-10; 1:1,000, #SC-5279, Santa Cruz), anti-SSEA4 (1:1,000, #414000, Life Technologies), anti–TRA-1-81 (1:1,000, #MAB4381, Millipore), anti-Cux1 (1:500, #ab54583, Abcam), anti-APP (6E10; 1:2,000, #803016, Biolegend), anti-human phospho-Tau at Thr231 (AT180; 1:1,000, #MN1040, Thermo Fisher Scientific), anti-total Tau (TAU-5; 1:20,000, #AHB0042, Life Technologies), anti–βIII-tubulin (TUJ1; 1:5,000, #MAB1637, Millipore), anti-GAPDH (1:10,000, GAPDH, Thermo Fisher Scientific), rabbit anti-cleaved Caspase-3 (Asp175; 1:1,000, #9661, Cell Signaling), guinea pig anti-DCX (1:2,000, #AB2253, Millipore), and chicken anti-MAP2 (1:5,000, #ab5392, Abcam).
Validation	All antibodies were validated by the manufacturer or published studies using western blot or immunocytochemistry analysis.

Eukaryotic cell lines

Policy information about cell lines

Cell line source(s)	Dr. Lawrence Goldstein (University of California, San Diego) generously provided one nondemented control-derived iPSC line (NDC1.1) and one AD patient-derived iPSC line (APP1.1) carrying APP gene duplication, both from male donors.
Authentication	The iPSC lines were generated and authenticated by the original publication (Israel et al., Nature, 2012).
Mycoplasma contamination	The iPSC lines were tested and confirmed to be negative for mycoplasma contamination.
Commonly misidentified lines (See ICLAC register)	No commonly misidentified cell lines were used.
